# Importance and Considerations of Antibody Engineering in Antibody-Drug Conjugates Development from a Clinical Pharmacologist’s Perspective

**DOI:** 10.3390/antib10030030

**Published:** 2021-07-26

**Authors:** Andrew T. Lucas, Amber Moody, Allison N. Schorzman, William C. Zamboni

**Affiliations:** 1 Division of Pharmacotherapy and Experimental Therapeutics, UNC Eshelman School of Pharmacy, University of North Carolina at Chapel Hill, Chapel Hill, NC 27599, USA; andrew_lucas@unc.edu (A.T.L.); aschorz@email.unc.edu (A.N.S.); 2 Carolina Center of Cancer Nanotechnology Excellence, UNC Lineberger Comprehensive Cancer Center, University of North Carolina at Chapel Hill, Chapel Hill, NC 27599, USA; amoody832@gmail.com; 3 Glolytics, LLC, Chapel Hill, NC 27517, USA

**Keywords:** antibody-drug conjugates, pharmacology, pharmacokinetics, mononuclear phagocyte system

## Abstract

Antibody-drug conjugates (ADCs) appear to be in a developmental boom, with five FDA approvals in the last two years and a projected market value of over $4 billion by 2024. Major advancements in the engineering of these novel cytotoxic drug carriers have provided a few early success stories. Although the use of these immunoconjugate agents are still in their infancy, valuable lessons in the engineering of these agents have been learned from both preclinical and clinical failures. It is essential to appreciate how the various mechanisms used to engineer changes in ADCs can alter the complex pharmacology of these agents and allow the ADCs to navigate the modern-day therapeutic challenges within oncology. This review provides a global overview of ADC characteristics which can be engineered to alter the interaction with the immune system, pharmacokinetic and pharmacodynamic profiles, and therapeutic index of ADCs. In addition, this review will highlight some of the engineering approaches being explored in the creation of the next generation of ADCs.

## 1. Introduction

Antibody-drug conjugates (ADCs) are one of the most complex biochemical agents to be produced this century as an advanced platform to deliver highly potent cytotoxic agents with guidance of an immunoglobin (IgG). While the idea of an ADC may seem simple, in over three decades of research there have been only ten clinically approved agents, the majority of which were approved in the last two years [1,2]. However, with cancer as the second leading cause of death in the United States, a significant effort has been given to the development of these agents as a method to overcome the toxicities of traditional systemic chemotherapies and deliver even more potent cytotoxic agents directly to malignant cells. Similar to the application of nano-technology to improve anti-cancer efficacy and reduce toxicity, this goal has been difficult to achieve with ADCs due to a seemingly narrow therapeutic index of the agents [3,4]. This is reflected in the significant number of ADC compounds that fail to reach late-stage registration trials and even more that do not translate beyond preclinical studies [1].

As ADC engineering appears to be in the beginnings of a boom, with three FDA approvals in 2019 and two more in 2020, along with several expanded indications for previously approved agents (Table 1), this platform has achieved a clinical validation that represents the evolution and convergence of numerous fields of study developed over the past three decades. In this review, we will discuss many of the factors and influences that need to be considered and shape the unique pharmacology (pharmacokinetics [PK] and pharmacodynamics [PD]) and interactions with the immune system of currently approved ADCs. We will also address some of the key challenges and ways clinicians and researchers have been attempting to improve the clinical efficacy of these agents.

## 2. Structural Considerations Affecting ADC Disposition

The technology surrounding ADCs is complex and fundamentally different from traditional small molecules therapies and even most non-conjugated therapeutic monoclonal antibodies (mAbs) [1,5]. As such, both the components that make up an ADC and a multitude of factors that influence their disposition based on their design must be considered and gives support to the difficulties and limited successes of this novel platform.

### 2.1. Antibody Selection

One of the first historical problems of ADCs, and all biologic agents, is the immunogenicity of the compound. To overcome this problem, many efforts have been made to design ‘humanized’ mAbs, containing murine complementary determining regions (CDRs) along with human variable regions. However, it was later realized that repeated administration, even of these chimeric antibodies, led to an immune response in patients. For this reason, techniques have been put in place that have led to the production of increasingly humanized antibodies, which unlike the previous designs, have a 90–95% human sequence and of the species used for immunization only maintain the regions determining complementary CDR. Another option has been to use fully human antibodies. Still, the chronic use of these antibodies could lead to adverse reactions in patients. Several industry guidance documents and white papers exist to help researchers understand how to evaluate the immunogenicity of individual compounds [6,7,8]. However, the development of novel engineered antibody formats (e.g., BiTE or bispecific antibodies) do not have a clear path on how to appropriately evaluate their immunogenicity. As the evaluation and engineering of antibodies against immunogenicity is an ongoing development, we direct readers to review the most current summary documents on the issue [6,7,9,10,11].

The most common mAb utilized in ADC development is the IgG1 subtype, similar to the majority of traditional non-conjugated (sometime referred to as ‘naked’) mAb therapies currently approved. This subtype has particularly been optimized to attempt to initiate antibody-dependent cellular cytotoxicity (ADCC) [12]. As ADCC and complement activation are not necessary components for ADCs to initiate their primary mechanism of action, such interactions occurring with ADCs could affect the therapeutic index (TI). For instance, ado-trastuzumab emtansine (Kadcyla) has a dose limiting toxicity (DLT) of thrombocytopenia, found to be due to a HER2-independent pathway of FcgRIIa (CD32a)-dependent uptake by megakaryocytes [13]. IgG2 isotypes offer the theoretical potential for more conjugation sites, allowing for an increased cytotoxic payload (binding to four disulfide bridges compared to two found in IgG1 or IgG4 isotypes) [14]. IgG2 antibodies also serve as being favorable as a therapeutic base due to their tendency to form covalent dimers, which helps enhance both the affinity and internalization of the antibody [15,16]. IgG3 isotypes have not been heavily used in therapeutic entities due to the significantly faster clearance (~3 times faster; in part due to increased proteolysis because of an elongated hinge region) compared to other IgG isotypes, despite being more efficient at inducing tumor cell lysis [15,17,18]. Some agents in preclinical studies have attempted to utilize this isotype with the rationale that the faster rate of clearance may allow for an improved toxicity profile, though the ADC may have to be administered more frequently. IgG4 isotypes are also being utilized in specialized functions due to their ability to form half antibodies (one heavy and one light chain) and swap fragments with other IgG4 units to generate hybrid bispecific-like compounds [19].

IgG isotypes also differ in their ability to initiate and/or support secondary immune functions, such as ADCC or complement-dependent cytotoxicity (CDC). IgG1 can support ADCC (a FcγR-mediated process) and CDC (a C1q-mediated process), but IgG2 and IgG4 isotypes are limited (or inefficient) in their secondary functions [20]. A clinical example demonstrates these differences as IgG1 brentuximab vedotin demonstrates better ADCC activity compared to IgG4 gemtuzumab ozogamicin, which has no effector functions [15,21,22]. IgG4 has been shown at times to support antibody-dependent cellular phagocytosis (ADCP), a process that relies on macrophages to devour tumor cells following antibody binding [23]. Along with the immune functions that antibody isotypes confer, there is preliminary evidence that suggests that modifying antibody isotype (e.g., IgM or IgA versus IgG antibodies) may make ADCs more targeted to the area of cancer, therefore limiting the potential for systemic toxicities [24].

Beyond the structure of the antibody, the other primary function of the antibody component of an ADC is to bind to its selected antigen moiety. These targets are currently focused around overexpressed or preferentially expressed molecules by tumor cells [25,26]. The antibody binding to its selected antigen on the tumor cell surface must also initiate the internalization of the entire surface complex to allow for intracellular delivery of the cytotoxic payload [27]. This is different from more traditional therapeutic monoclonal antibodies as the antibody itself is not required to have functional activity of its own (such as initiating ADCC). The majority of current FDA-approved ADCs are designed to target an antigen overexpressed on tumor cells. However, to increase the effectiveness of a single ADC agent among several cancer types, an emerging area of interest has been to target antigens related to the tumor stroma (discussed in more detail later in this review) [28]. Such a method can expand the therapeutic utility of an ADC beyond select groups of patients which are antigen-positive.

Another consideration is that the epitope of the antibody can also affect accessibility, so the type of antibody format utilized could impact antigen binding. This is exemplified by Samsudin et al., who modelled how hexamers of pertuzumab IgM antibodies were capable of utilizing all of their variable regions to bind HER2, but trastuzumab IgM becomes hindered by steric clashes due to the globular conformation of HER2 [29]. Similar steric observations were observed when pertuzumab IgM was compared to pertuzumab IgG. These observations were confirmed in vitro by inhibiting cell proliferation of HER2-expressing cell lines in culture [29].

### 2.2. Payload Options

Compared to therapeutic mAbs, ADCs are conjugated with highly toxic compounds. The potency of these compounds (commonly with an IC50 in the nanomolar to picomolar range) would preclude their use as an intravenously administered therapy due to the extreme risk of potent toxicities [30]. On the other hand, in theory, only a small amount of the drug would need to be delivered to the interior of a tumor to result in efficacious effects. Due to the large breadth of payloads explored, we highlight a few recommended reviews to provide a gauge of the current field and discuss the general classes of agents being employed [2,31].

**Figure 1:**
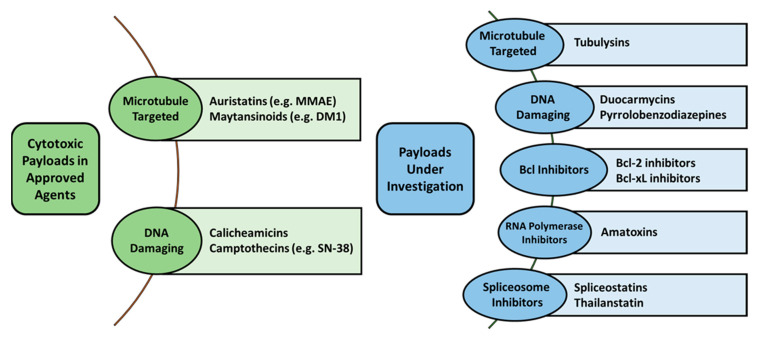
Overview of cytotoxic payloads conjugated to approved ADCs and ADCs in development.

All of the clinically approved ADCs and the majority of ADCs in development carry cytotoxic payloads that fall into one of two categories: anti-mitotic agents or DNA-binding agents (Figure 1). The first of these categories, anti-mitotic agents, is made up of three classes of agents: auristatins, maytansinoids, and tubulysins. Auristatins are synthetic agents that act by blocking tubulin polymerization thereby halting the cell cycle and triggering apoptosis [2]. The most common example an auristatin is monomethyl auristatin E (MMAE) and is the most common payload of the clinically approved ADCs, including brentuximab vedotin, Polatuzumab vedotin, and Enfortumab vedotin. Maytansinoids are the next most common utilized agents among investigated ADC agents, acting in a similar fashion to auristatins by inhibiting tubulin polymerization to result in mitotic arrest [2]. An example of a maytansinoid agent is emtansine or mertansine (i.e., DM1) which is utilized on ado-trastuzumab emtansine and ravtansine (i.e., DM4) is another compound used on late-stage ADCs such as anetumab ravtansine [2]. Finally, tubulysins are a more novel anti-mitotic peptide agents with picomolar potencies meant to inhibit microtubule polymerization and is theorized to bypass the efflux pumps that affect other anti-mitotic agents (e.g., DM1) [17].

The second category of agents utilized in ADCs, DNA-binding agents, encompasses a large population of different types of agents: calicheamicins, duocarmycins, camptothecins, anthracyclines, and pyrrolobenzodiazepines (PBD) [2]. Calicheamicins are a type of anti-tumor antibiotic, similar to doxorubicin, that bind the minor groove of DNA to interrupt the process of DNA replication [32]. A modified form of this agent (N-acetyl-calicheamicin; i.e., ozogamicin) is utilized in the ADC gemtuzumab ozogamicin and inotuzumab ozogamicin. Duocarmycins act in a similar manner to calicheamicins, through also causing irreversible alkylation of DNA that disrupts nucleic acid structures and affects the overall DNA structure [2]. However, none of these agents have yet made it to late-stage clinical trials. Anthracyclines are a common class of anti-tumor antibiotic, exemplified by doxorubicin and daunorubicin, which intercalate into DNA to inhibit DNA synthesis [2,33]. While first-generation ADCs attempted to utilize doxorubicin as a cytotoxic payload, the increase in hydrophobicity and lack of sufficient delivery to exert a potent anti-tumor effect limited their further translational use. However, with advances in higher drug-loading preparations and novel linker strategies (e.g., PEG chains) to conjugate large quantities of doxorubicin to an individual ADC, an increase in their evaluation as an ADC payload has grown in the past years. PBDs are another type of anti-tumor antibiotic that work by forming dimers which in turn allows the compound to crosslink to opposing DNA strands [17]. These agents have been heavily utilized in early ADC development and at least ten ADCs have entered registration trials, suggesting these agents show high clinical promise. However, none of these agents have yet to obtain FDA approval. Camptothecin analogs, such as SN-38, have begun to be evaluated once again now that higher drug-antibody ratio (DAR) agents are able to be synthesized with favorable PK profiles (e.g., IMMU-132/sacituzumab govitecan has a DAR of 8) [17]. SN-38, the active form of irinotecan with ~3 times greater potency, acts as a topoisomerase I inhibitor. A newer analogue, DX-8951, has also begun to enter early-stage trials and has demonstrated activity against multi-drug resistant cells in vitro [34].

In addition to the warheads discussed above, several promising new compounds are beginning to be utilized in preclinical development. One such class is an improvement on PBDs, termed indolinobenzodiazepine pseudodimers (IGNs), and are more potent than PBDs [35]. These IGNs can work either in a monomeric or dimerized form to act via DNA alkylation or DNA crosslinking, respectively, and the monomeric form carried an improved safety profile in comparison to PBDs or dimerized IGNs [35,36]. While malignant cells are typically not responsive to apoptotic signaling, a mechanism which can be done in part by overexpression of Bcl2, the use of Bcl-xL inhibitors have been developed to reinvigorate these apoptotic signaling deficiencies. EGFR-targeted ADCs carrying Bcl inhibitor payloads have shown promising results in preclinical models when used in combination with docetaxel [37]. Dysregulation in RNA splicing is another mechanism reported in several cancer types [38]. Spliceostatins have shown promising results in panels of malignant cell lines and thailanstatins have been conjugated to HER2 targeting mAbs to provide low nanomolar potency in similar panels of cell lines [39,40]. With this advent into RNA and other transcription inhibitors, the development of RNA polymerase inhibitors has become a new area of interest. Amatoxins, peptides that are selective in their inhibitors of RNA polymerase II, have been successfully conjugated to HER2 mAbs and demonstrated picomolar potency [41]. Beyond their potency, amatoxins are also unique to ADCs as they are comparably more hydrophilic (which also results in reduced aggregation) and the small size of the drug allows them to accumulate in tumors but be quickly excreted by the kidneys if released in circulation or normal tissues [32,42].

### 2.3. Linker-Drug Stability

Chemical linkers are a fundamental component to attach a cytotoxic payload to a mAb and derive the efficient delivery of the payload but is also a key factor that describes the toxicity of an ADC. These linkers are comprised of various functional groups in order to conjugate the cytotoxic payload to the mAb as well as ideally controlling the distribution and release of the payload into the targeted cell [15,43]. This matter of release from the antibody carrier can cause potent off-target toxicities if released before entering the tumor and instead injuring healthy tissues. As these chemical linkers are an essential component in influencing the PK and TI of an ADC, the linker needs to be stable enough to allow for efficient drug release, but only at or in the targeted cells. The chemical linkers used currently are broadly grouped into two categories: cleavable and non-cleavable linkers. As there are 100+ patents on various forms of chemical linkers, we refer readers to key review articles and discuss the core properties to consider below [44,45].

By definition, cleavable linkers are characterized by a cleavage site located between the cytotoxic payload and the mAb. This cleavage can occur by physiological stimuli and by multiple different mechanism based on the specific linker chemistry: acid-labile hydrolysis, enzymatic, or reductive being the most employed [41,45,46]. Acid-labile linkers, commonly containing hydrazones, are sensitive to the acidic pH environment of a lysosome and are employed by inotuzumab ozogamicin. These linkers typically show the best stability at a neutral pH, such as in circulation; however, extravascular cellular compartments can be slightly acidic and have been shown to cause premature release of the payload in clinical studies. Enzymatic cleavage, usually designed to be due to a lysosomal protease (e.g., a valine-citrulline bond), is employed by brentuximab vedotin. Finally, reduction of disulfide bonds is carried out by glutathione within the cytosol. This mechanism is used by gemtuzumab ozogamicin, though its previous design (before it was first removed from the market in 2010) employed an acid-labile hydrazone linker. However, several ADCs are also in development that use this linker type [47].

On the other hand, a non-cleavable linker is meant to remain stable and prevent the release of the cytotoxic payload while in circulation or in extravascular spaces and only releases the cytotoxic cargo once it is internalized and broken down in the lysosome [45]. These linkers commonly employ thioether chemistries that require the proteolytic breakdown of the antibody carrier before the cytotoxic payload can be ‘released’ [45]. The use of non-cleavable linkers on an ADC are thoughts to provide an improved therapeutic index due to the improved plasma stability. However, new development and designs of certain cleavable linker chemistries have begun to challenge this thought by improving plasma stability [48]. Due to this method of release, the cytotoxic agent may also form a heterogeneous population of tagged payloads depending on the length of the degradation product still attached to the payload. Therefore, it is important to ensure the linker conjugation to the payload does not block or interfere in the mechanism of action of the cytotoxic agent.

### 2.4. Drug-Antibody Ratio (DAR)

The DAR, or the number of compounds conjugated onto an individual ADC molecule, is one of the most critical factors in determining the efficacy of an ADC. However, the optimal DAR has yet to be determined across all ADCs, though this is likely due to the fact that multiple variables also need to be incorporated along with the DAR to achieve the greatest clinical outcomes for an individual agent. It can be surmised that a sufficient number of cytotoxic molecules needs to be attached to provide an adequate anti-tumor effect; though if too many molecules are attached to the ADC this can lead to increased toxicity, have increased recognition by the immune system, and alter PK disposition and faster clearance [49]. For example, the conjugation of MMAE or doxorubicin in high DAR configurations commonly results in increased aggregation and alteration in PK properties, which is often contributed to the increased hydrophobicity of the overall ADC [49,50,51].

**Figure 2:**
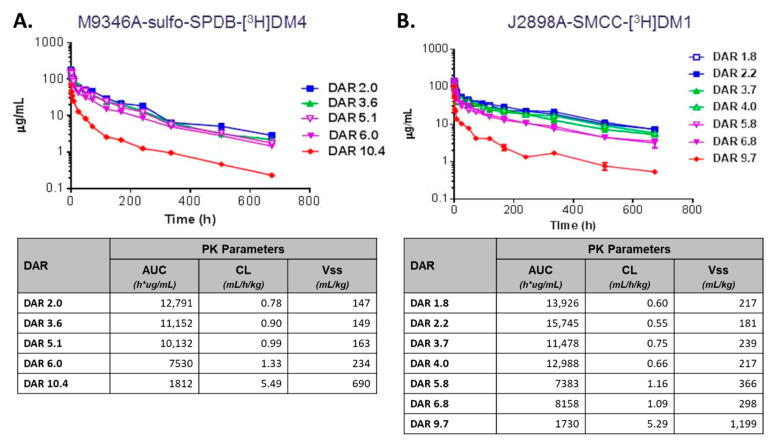
Pharmacokinetic studies evaluating ADC concentrations using radiolabeled ADCs. Clearance of M9346A–sulfo-SPDB–[3H]DM4 (**A**) and J2898A–SMCC–[3H]DM1 (**B**) conjugates from plasma of CD-1 mice. All agents were injected iv as a single 10 mg/kg dose. Concentrations of total ADC were measured by counting the radioactivity in plasma arising from the tritium label on the maytansinoid. These findings suggest that maytansinoid conjugates, regardless of linker type, with drug–antibody ratios (DAR) ranging from 2 to 6, have a better therapeutic index than conjugates with very higher DAR (>9). Reproduced and altered with permission from Sun et al. Bioconjug. Chem. 2017, 28, 1371–1381 [52].

There have been numerous studies on the effects of DAR on the pharmacologic properties of ADCs. A key study of note was performed by Sun et al., where a sequence of ADCs was conjugated with either DM1 or DM4 in a DAR ranging from two to fourteen using both a cleavable and non-cleavable linker [52]. This series of ADCs were then evaluated in preclinical mouse models for ADC stability, PK disposition, and efficacy. These studies show how ADCs with a higher DAR (mean DAR 10) display faster rates of distribution to tissues and blood clearance along with a decrease in efficacy (Figure 2) [52]. However, ADCs with a DAR of less than six commonly displayed similar PK properties and overall greater tolerability compared to higher DAR preparations. A similar study using an anti-CD30 MMAE-conjugated ADC demonstrated similar results of increased clearance and reduced efficacy with increasing DAR (evaluated from DAR 2 to DAR 8) [49].

DAR used to be a difficult factor to evaluate in the clinic as the production of any single early-generation ADC product would result in heterogeneity, so DAR is reported as a majority or mean DAR [53]. This is driven due to the number of conjugation sites on the ADC, such as lysine (as in ado-trastuzumab emtansine) or cysteine (as in brentuximab vedotin) residues accessible on the surface of the ADC. It is important to note that all of the FDA approved ADCs employ a conjugation chemistry that binds to random native lysine or cysteine residues (with exception to moxetumomab pasudotox-tdfk, which is a novel recombinant linker-less ADC) [54,55,56]. However, in the past few years, the development of site-specific conjugation methods to control drug loading and resulted in the emergence of a more homogenous ADC product. In fact, the proportion of ADCs entering clinical trials that use site-specific conjugation technologies has been steadily growing since 2011, and with almost all new ADCs entering clinical trials using these methods in 2020 [57]. In general, these site-specific conjugations follow one of three strategies: engineered cysteine residues, enzymatic-based conjugation, or the use of unnatural amino acids [58]. The overall goal of using site-directed conjugation is to generate a more homogenous mixture of ADCs, which in turn should help normalize and potentially improve the PK variability while reducing toxicity. A DAR of two to four had been considered optimal, but recently approved ADCs using their site-specific conjugation methods have shown the advancement in higher DAR preparations (e.g., trastuzumab deruxtecan had a DAR of 8) [49].

### 2.5. Surface Modification

Taking guidance from the history of nanoparticle development, PEGylation is one of the first mechanisms that attempted to modify the disposition of ADCs to improve the pharmacologic profile. The basis for the addition of PEG polymer is useful to improve the solubility of pharmacologic agents, prolong circulation within the body, and to reduce immunogenicity to the antibody portion of the ADC [59,60,61,62]. However, due to the potential of steric hinderance associated with the length of the PEG polymer chain, conjugation sites on ADCs need to be carefully considered and should not interfere in antigen recognition or antigen affinity. Because of this, PEGylation has mainly been used to serve as a way to increase the DAR of an ADC by acting as a linker, though this mechanism is still being tested mainly in preclinical models. One of the best studies evaluating the effect of PEG polymer chain length on the PK of an ADC was performed by Burke et al., where Sprague-Dawley rats were administered a 3 mg/kg dose IV x 1 of MMAE ADC (with a fixed DAR of 8) with variations in the chain length [59]. As expected, the addition of the PEG chain and an increasing PEGylated chain length caused reduced clearance of the ADC [59]. Furthermore, PK parameters were only minorly affected with chain length between eight and twenty-four PEG polymers [59].

Another engineered modification to ADCs that has regularly been employed in approved ADCs is controlled glycosylation. While glycosylation is a natural post-translational modification that occurs within all eucaryotic cells, the amount and locations of these additions results in dramatic alterations to the PK and PD disposition of an ADC [63,64,65,66]. An example of a glycoengineered ADC that exhibits these effects is ado-trastuzumab emtansine, which has terminal sialic acids, as N-linked glycosylation has been shown to promote prolonged systemic circulation time [66,67]. It is important to note that there is conflicting data on the effects of glycosylation within the Fc region and the effects on clearance rates, but it has been accepted that higher levels of mannose-5 glycan result in faster ADC clearance due to binding to mannose receptors [63,68]. Nonetheless, the analysis of differing glycosylation states is complicated due to heterogeneity in these modifications (both the type and amount) during production and the inability to generate compounds that can be directly compared.

## 3. Biological Interactions Affecting ADC Pharmacologic Disposition

### 3.1. Target Expression and Affinity

Target antigen expression levels are a key parameter in predicting the benefit of ADCs to a patient where the higher the cell surface target antigen density, the more ADC can be taken up and metabolized by the cell to release the cytotoxic agent. The appropriate selection of a target for an ADC is extremely complex and complicated by the potential limited targets on the cell surface and the various mechanisms of internalization that can be inefficient [69]. Traditionally ADCs have been designed to target a surface antigen that is overexpressed on the surface of malignant cells while not (or minimally) expressed on normal cells or tissues [15,17]. This indicates that a patient selection strategy based on high antigen target expression levels would aid in identification of the appropriate population for treatment with ADCs [70]. In hematologic malignancies, surface receptors marking malignant cells are commonly homogenous, allowing ADCs to target these cells for destruction—and commonly termed “lineage ablation” [71]. On the other hand, overexpressed antigens marking solid tumors are not tumor-specific but tumor-associated, making the therapeutic index more difficult to determine in solid tumors compared to hematologic malignancies. In addition, the relative expression of target antigen on tumor cells versus normal tissue must be evaluated to determine the therapeutic index of an ADC, especially in solid tumor indications [72].

Experimental evidence would suggest that a correlation should exist between surface target antigen presentation and an ADC’s efficacy. The amount of antigen necessary for an ADC to be effective will be target antigen specific [73]. Preclinical models allow assessment of expression levels in the whole body, evaluation of antibody access, and confirm internalization of the cytotoxic agent in tumor tissue versus normal tissues. Sharma et al. performed a quantitative in vitro investigation of the effect of antigen expression levels on ADC exposure within four cancer cell lines and showed a strong linear relationship between ADC exposure and target expression levels [74]. The limitation in using preclinical animal models is that most preclinical host animals do not express the humanized target antigen, so difficulty exists in replicating the preclinical activity in clinical trials [71].

Clinical studies of target antigen expression levels also show a strong correlation between elevated expression levels of the target antigen and clinical outcome. For example, for the FDA approved drug ado-trastuzumab emtansine, the phase III clinical trial suggested that patients with increased breast tumor HER2 expression levels demonstrated improved progression-free survival and overall survival, median and low expression of HER2 resulted in median and lower overall survival, respectively [75]. Along with a simple correlation of expression level and ADC exposure, there is a minimum threshold or cutoff value of antigen expression as a prerequisite for ADC efficacy. It has previously been estimated that a minimum of 104 to 105 antigens per cell are necessary to ensure the delivery of sufficient ADC to induce cytotoxic cell death [25,76]. This cutoff level varies greatly between target antigens and also depends on other factors including internalization rate, binding affinity, and sensitivity to the payload [53]. Due to this limited target presentation and taking into account the average DAR of early ADCs, a very small amount of the cytotoxic payload of ADCs are actually delivered into cells. Thus, highly potent agents are necessary for and may provide explanations why traditional cytotoxins (e.g., methotrexate, doxorubicin) did not provide robust clinical results in the first ADCs [17].

In fact, it has also been proposed that sensitivity to the cytotoxic payload is more important that antigen binding. A study performed in cell culture evaluated an anti-CD22-DM1 agent against a panel of non-Hodgkin lymphoma (NHL) cell lines (*n* = 18). The results of this screening panel demonstrated that the specific cell sensitivity to DM1 was more predictive of response compared to the surface expression of the target antigen [77]. This would suggest the selection of patients based solely on target expression may not guarantee efficacy, and additional tumor and/or non-tumor sensitivities and markers may be necessary to appropriately select the best patients for a given ADC therapy.

Discrepancies in the use of target expression as a means to predict outcomes have been complex and inconsistent for ADCs. A well know example is presented in the clinical development and progression of registration trials of rovalpituzumab tesirine. In the initial phase I studies, clinical response (using an endpoint combining complete and partial response) was determined to only exist in patient expressing high levels of DLL3 [78]. Once progressed into phase II evaluations, there was no significant difference in outcomes observed between patients with DLL3-high tumor versus a more general DLL3-positive tumors, even among different cancer types [79,80]. Ultimately, even the phase III study of rovalpituzumab tesirine in small cell lung cancer was placed on hold when the comparator arm topotecan demonstrated improved overall survival [81]. Similar inconsistencies between studies have also been documented in the phase I studies of mirvetuximab soravtansine (a folate receptor alpha targeting ADC), even within the same disease group [82,83,84]. So far, there has been no explanation provided to justify the rationale of why these inconsistencies occurred, but it does highlight the ongoing challenges in understanding the drivers and variance of target expression and how to translate this into selection of the optimal agents for patients and to improve overall response.

While the above clinical examples provide evidence of target expression in tumors not being able to predict outcomes, other more harmful examples exist where significant toxicities may present if ADCs bind targets outside of tumors. The distribution and exposure of ADCs to normal tissue expressing the target antigen has demonstrated gastrointestinal toxicity leading to death in patients treated with BAY794620 that targeted CA9 antigen and fatal exfoliate of skin toxicity in patients treated with bivatuzumab mertansine that targeted CD44v6 antigen [85]. Thus, a target antigen should be selected that exhibits a high level of tumor-specific expression and minimal expression in normal tissues or tissues that are expendable (e.g., certain reproductive tissues, adipose) or able to regenerate. For example, Rituximab targets the CD20 antigen that is expressed on B cells and kills both malignant and normal B cells; however, depletion of B cells is not a major safety issue [86].

### 3.2. Non-Specific Endocytosis

Endocytosis is an important process that affects the distribution of macromolecular complexes, such as therapeutic mAbs and ADCs [87]. In general, endocytosis can be grouped by the size of the endocytic vessel (microscale for <200 nm; macroscale for 0.2–10 uM) and the type of solute internalized (phagocytosis for internalization of particulate; pinocytosis for internalization of soluble molecules) [88,89,90]. Typically, microscale endocytic mechanisms, including clathrin-mediated and caveolin-mediated endocytosis, are the predominant form of target-dependent internalization by ADCs [91,92]. These processes are initiated by the binding of ligands to specific membrane receptors, resulting in a cascade of signals leading to endocytic vesicle formation [93,94]. Regardless of the method of internalization, endocytized materials are then subjected to intracellular processing and catabolism, ultimately leading to degradation within the lysosome.

Internalization of the ADC based on endocytic uptake of the antibody into the cell is important for optimal efficacy. Regardless of the mechanism of endocytosis (macro- or microscale), these mechanisms contribute to normal and cancer cells internalizing ADCs. However, while it is clear that the rate of endocytic mechanisms among normal cells and tissues, macropinocytosis, caveolar-dependent endocytosis, and phagocytosis are believed to be the most important processes [95]. Among these cells, immune cells that specialize in endocytosis (e.g., monocytes and macrophages) perform these processes as their primary role and thus display increased rates of endocytosis compared to other non-malignant cells and tissues. These immune cells have also been implicated in several studies to play a major role in the clearance of immunoconjugates—including ADCs [96]. Endothelial cells also display a higher rate of endocytosis compared to the majority of normal cells, especially of macromolecular complexes, due to increased opportunities related to their positioning between the vasculature and interstitial compartment. Understanding the differences in the rates of presentation and rates of endocytosis of various normal cells is an important step in understanding the contribution of non-specific endocytosis towards ADC PK disposition and toxicities.

The two physiochemical properties of ADCs that have been demonstrated to influence non-specific endocytosis are the surface charge and hydrophobicity of the linked compound [97]. The cell membrane of mammalian cells contains several negatively-charged groups on its surface, thus attracting positively-charged compounds [98,99]. Prior studies investigating the increase in positive-charge of mAbs after linking drugs has shown both an increase in plasma clearance and tissue distribution [100]. While similar studies have not been performed in ADCs, it stands to reason that changes in the net ADC charge will also influence an ADC’s rate of non-specific endocytosis, making charge modification an important physiochemical parameter. However, these modifications will also need to be balanced against changes to target antigen recognition that affect altering antigen-dependent uptake of ADC by tumor cells and its efficacy. Hydrophobicity is primarily influenced by multitude of drug-linker combinations employed, especially with ADCs that have a higher DAR of hydrophobic payloads [101,102]. Increased hydrophobicity can further lead to aggregation of ADCs, which also leads to increased non-specific clearance due to increased recognition by endothelial cells and immune cells (e.g., monocytes and macrophages) [101,102]. These data suggest that altering the hydrophobicity associated with the conjugated payload is a key factor in the design and optimization of ADC disposition and potentially providing a feasible path to modulating the therapeutic index of an agent.

### 3.3. Antigen Targets Resistant to Internalization

Resistance to ADCs can be present at the start of primary treatment or evolve after treatment or continued exposure to the drug. One possible mechanism of resistance is a change in the level of the target antigen. In one study, after multiple exposure cycles to an anti-HER2 trastuzumab-maytansinoid ADC that was structurally similar to ado-trastuzumab emtansine resulted in breast cancer cell lines becoming resistant to ado-trastuzumab emtansine. A decrease in the levels of the HER2 protein expressed was also observed in this experiment several months after the start of treatment [103]. Tissue biopsy samples obtained from patients with anaplastic large cell lymphoma (ALCL) treated with brentuximab vedotin at the time they relapsed or progressed showed varied expression of CD30 on ALCL cells [104]. On the contrary, high antigen expression may also reduce effectiveness of ADCs. In one example, gemtuzumab ozogamicin, which targets CD33, shows limited penetration in bone marrow when there are high CD33 antigen loads in peripheral blood [105]. Ligands of antigens can also change ADC sensitivity, such as neuregulin, which promotes heterodimerization of HER2 with HER3, and HER4 can impair the efficacy of ado-trastuzumab emtansine [106].

Following endocytic uptake, the ADC needs to reach the lysosome to release the cytotoxic agent by chemical or enzymatic cleavage. Subsequently, the proteolytic activity within the lysosome needs to be sufficient for degradation of the ADC. Chronic exposure to ADCs, including ado-trastuzumab emtansine, can cause a decrease in lysosomal acidification and slow proteolytic turnover resulting in reduced linker cleavage and payload release [103,107,108]. This mechanism of resistance results in reduced efficacy of ADCs where lysosomal acidic proteases allow degradation of the ADC. In some cases, such as ADCs with noncleavable linkers, lysosomal membranes are impermeable to the catabolites needed to release the linker-cytotoxic agent attached to an amino acid residue, therefore requiring transport out of the lysosome and into the cytoplasm. Resistance due to transport to the cytosol is caused by SLC4683, a lysosomal membrane protein that inhibits the potency of multiple non-cleavable linker ADCs [109]. The subsequent elimination of the cytotoxic agent after release from the cellular cytoplasm by the ATP-binding cassette (ABC) transporters is another mechanism of ADC resistance due to many of the cytotoxic agents being substrates of ABC transporters [110,111,112]. Upregulation of ABCB1 or multidrug resistance protein 1 (MDR1) has been shown to eject payload that has entered the cytoplasm demonstrating drug resistance to both gemtuzumab ozogamicin and inotuzumab ozogamicin [113,114,115]. Loganzo et al. reported that inhibition of upregulated ABC transporters causing ADC resistance allowed restoration of ado-trastuzumab emtansine sensitivity in HER2+ gastric cancer cells, which highlights the potential for alternate therapies to combat this drug resistance [103].

### 3.4. Non-Antigen Receptor-Mediated Uptake

Target-independent uptake (i.e., binding of antibody not to the target antigen) is mediated by a host of different receptors that recognize various parts of the Fc backbone of an antibody. While Fc-mediated functions are not considered necessary for ADC functionality, non-antigen receptor-mediated internalization into non-malignant cells may lead to toxicity and into innate immune cells may lead to higher clearance and reduced efficacy. A key example is how FcγRs on circulating and tissue-based innate immune cells serve as a major pathway for the clearance/elimination of mAbs and ADCs.

FcɣRs plays a primary role in target-independent uptake mechanisms as these receptors are the pivotal link between the innate and humoral immune response [1]. These receptors are also part of the initiation of various antibody-mediated effector function such as ADCC, CDC, ADCP, and release of immune regulating cytokines (e.g., IFNɣ and TGFα) [116,117]. As these receptors are not responsible for target-related efficacy, understanding the expression pattern on normal and immune cells can help predict potential ADC toxicities. The roles and functions of FcɣRs and the neonatal Fc receptor (FcRn) on immune cells have been reviewed extensively [1,118,119,120,121]. The binding affinity of the ADC to FcRn effects PK biodistribution due to the expression of FcRn throughout the body in kidneys, liver, muscle, skin, spleen, fat cells, and placenta. It is important to note that there is contradicting evidence on how Fc mutations affect the interaction between FcRn [122,123,124,125,126]. Some data demonstrate a direct relationship between changes to in vivo clearance to certain mutations, but other mutations results in no change in clearance, the specific mutation (or perhaps total number of mutations) may dictate the total change in clearance of a particular mAb agent. Surprisingly, certain minor Fc mutations appear to also affect an antibody’s ability to bind to it select antigen. Su et al. performed a study analyzing the effects of the Fc region of IgA variants of pertuzumab (anti-HER2) [127]. This group discovered that Fc mutations, specifically heavy chain mutations to conserved cysteine residues C266Y/H317R in the IgA1 and C253Y/H304R in IgA2 antibody, reduced overall mAb binding to HER2 in vitro.

In addition to immune cell related FcɣRs, another key site of these receptors in on the surface of platelets, specifically FcgRII. While endocytic internalization may not be an issue with platelets, binding of ADCs to the surface of platelets leads to an aggregation/clearance/sequestration of drug from circulation [128,129]. This has been observed in patients with an acute drop in platelet counts (usually over the course of 5 to 7 days). In addition, this is seen in hepatotoxicity of ADCs conjugated with calicheamicins in liver sinusoidal endothelial cells (LSECs). While the exact mechanism of uptake of ADCs by LSECs which results in this specific hepatotoxicity is unknown, the mechanism is thought to be target-independent as ADC targets eliciting this effect are found on several other cell types (e.g., CD33) or are not found in the liver (e.g., CD22) [130].

**Figure 3:**
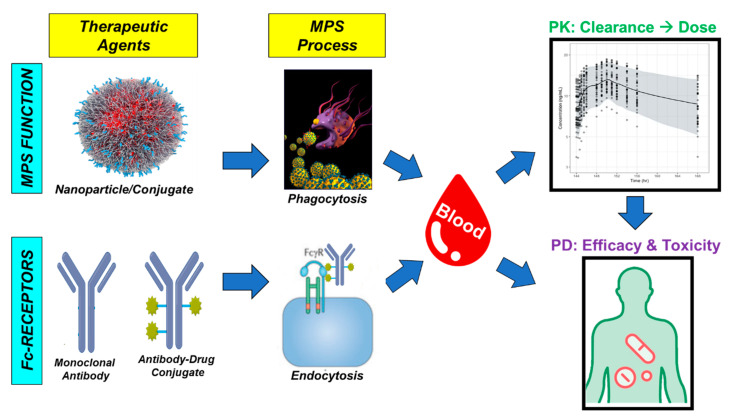
Clearance of nanoparticles (NPs) and mAb/ADC by MPS cells via phagocytosis and FcɣR endocytosis, respectively, and the effects on PK blood/serum exposure, efficacy, and toxicity.

The expression profile of FcɣRs in various tissues further offers PK considerations for antibody-based agents due to the potential to bind the Fc-region of the ADC. Similar to ‘naked’ therapeutic mAbs, the mononuclear phagocyte system (MPS), which is part of the innate immune system (IIS), serves as a natural mechanism of clearance for ADCs via their FcɣRs [119,131,132] (Figure 3). The pharmacologic disposition of ADCs are very similar to that of nanoparticles where the MPS drives distribution, clearance, and activation. The clearance of mAbs is highly variable between patients where the variability in clearance of trastuzumab is approximately 43% [133]. The distribution of both nanoparticles, mAbs, and ADCs are shown to be higher in MPS organs including the liver, spleen, and lung [134,135,136,137]. However, the differing isotypes of IgGs carry different affinities to FcɣRs [118]. Abuqayyas et al. has suggested that mAb PK (and thus ADC PK) are not markedly influenced by FcɣRs [138]. In a preclinical mouse study, a IgG1 agent was administered at doses between 0.04–0.4 mg/kg in non-tumor bearing C57BL/6 wild-type and knockout (FcγRI/RIII or FcγRIIb) mice and plasma concentrations were evaluated. The clearance of mAb from plasma were similar for all evaluated doses and in all animal cohorts. However, it is important to note that these IgG1 doses were 100- and 250-fold lower than the allometrically-scaled “therapeutic doses” of other IgG1 antibodies, such as pertuzumab (30 mg/kg) and trastuzumab (100 mg/kg), respectively [139,140]. Thus, this study’s conclusions that there was a low likelihood that FcγR expression affected plasma PK parameters could be due to the use of “micro-doses” in this study as the PK disposition of mAbs is dose dependent. Thus, further studies need to confirm FcyR effects on ADC PK. Interestingly, a higher exposure of IgG1 agent was observed in the liver (an MPS organ) in FcγRI/RIII knockout mice compared to wild-type mice [138]. Thus, even at “micro-doses” of mAbs, FcɣR expression does display a noticeable effect on tissue distribution.

**Figure 4:**
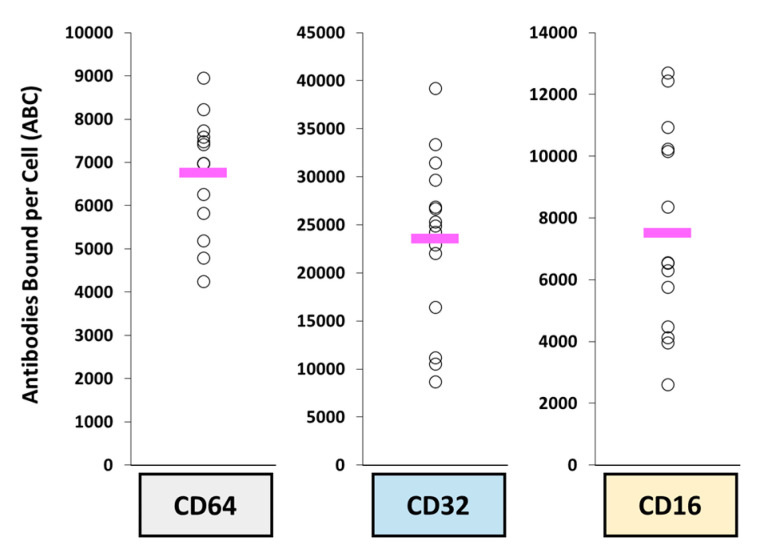
Variability in FcɣR expression on circulating monocytes (CD14+) in the blood of patients with metastatic breast cancer (mBC). CD64, CD32, and CD16 FcɣRs were evaluated in each patient. Open circles and bars represent individual patient results and mean, respectively. This high interpatient variability in the expression of FcɣRs on MPS cells in blood in consistent with the interpatient variability in the PK of mAbs and ADCs [143].

Finally, it has been noted that the PK variability of both naked mAb and ADC agents appears to be similar to the variability in FcγR expression on circulating MPS cells in blood of patients. Prior studies have previously reported the high variability in the function of these phagocytic cells and its relationship with the variable PK observed with nanoparticle agents [141,142]. A follow-up study by one of these groups evaluated the inter-patient variability in FcɣRs expression on MPS cells in blood in patients with metastatic breast cancer (*n* = 15 patients) [143]. The interpatient variability in CD64, CD32, and CD16 were 2.9-fold, 4.8-fold, and 7.8-fold, respectively (Figure 4). This fold difference in expression is similar to previously reported variability in plasma PK parameters (e.g., Cmin, AUC) of mAbs and ADCs from clinical trials, including rituximab [144,145,146], ado-trastuzumab emtansine [73,147,148], and inotuzumab ozogamicin [149,150,151]. Thus, similar to the effects of variability in MPS function on the PK and PD of nanoparticles, the inter- and intra-patient variability in MPS function and FcɣRs may be responsible for the PK and PD variability of mAbs and ADCs and thus needs to be further evaluated.

It has also been suggested that glycosylation on the Fc region of IgGs can be recognized by a group of receptors called C type lectin receptors (CLRs) [152,153]. These receptors are generally classified as Type I or Type II. Type I CLRs are calcium-dependent and include receptors such as CD206 (macrophage mannose receptor); Type II CLRs can be calcium-dependent (e.g., Dectin 2) or independent mechanisms (CLEC9A) [154]. CLRs are typically found on the cell membrane of myeloid immune cells and various other antigen presenting cells (APCs), though these cells have been shown to generate soluble/secreted versions of these receptors [154]. While CLRs have not been directly demonstrated to contribute to off-target ADC toxicities, mannose receptors specifically have been implicated as a potential mechanism of hepatotoxicity of ADCs [153]. Several ADCs have reported toxicities that could not be explained by target-independent antigen expression [155], and the common factor among them is an insult to LSECs [156,157]. The mannose receptors on LSECs are important in the clearance of glycoproteins and mannosylated antibody-enzyme fusion proteins, further supporting the importance of these cells in the clearance of biotherapeutic compounds and ADCs [158]. Kupffer cells also express the mannose receptor and may play an important role in non-specific uptake of ADCs, so the results with LSECs may translate or synergize with these macrophages [96,159]. Ultimately, this means the glycosylation profile of an ADC is an important aspect to evaluate in order to estimate the degree of CLR interaction.

## 4. Bioanalytical Methods to Evaluate Novel Engineered ADC Disposition

**Figure 5:**
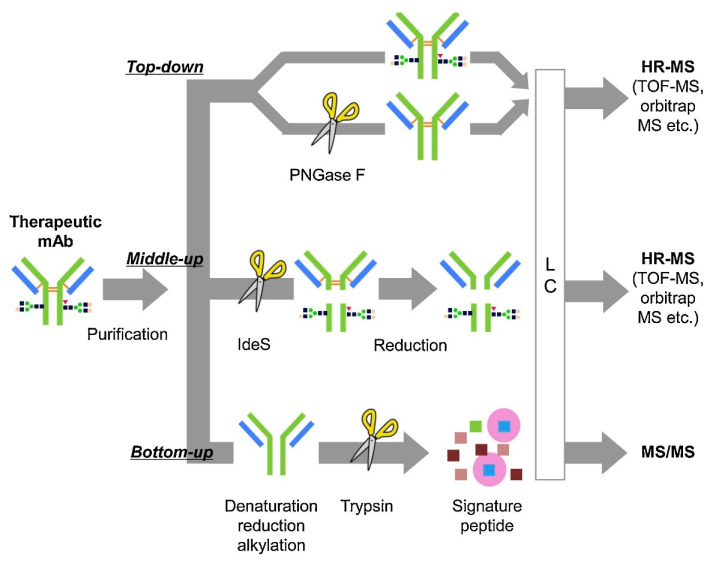
A workflow of bioanalysis for therapeutic mAbs by bottom-up, middle-up, and top-down approaches. Reproduced with permission from Todoroki et al. J. Pharm. Biomed. Anal. 2020, 179, 112991 [161]. Abbreviations: HR-MS, high resolution mass spectrometry; LC, liquid chromatography; MS, mass spectroscopy; MS/MS, tandem mass spectroscopy; TOF-MS, time of flight mass spectrometry.

The size and heterogeneity of ADCs present a bioanalytical challenge in drug development and determination of pharmacologic disposition. Specifically, accurate and robust methods are required to perform the quantitative and qualitative characterization of the antibody, the payload, and its linker (cleavable/non-cleavable), which are all critical in the efficacy of a given ADC [160]. Ligand-binding assays (LBAs), such as ELISAs, have been traditionally used to measure ADCs due to their high throughput and sensitivity [161]. In the past decade, methods based on liquid chromatography coupled with mass spectrometry (LC-MS) are being relied upon as an alternative and complementary method to LBAs for measurement of ADCs due to their sensitivity and specificity. Bioanalytical LC-MS based methods require enrichment of the ADC from the plasma/serum by immunoaffinity capture prior to analysis. This can be accomplished using the antigen as a probe to isolate the specific ADC via the Fab region or using Protein A/G beads to pull down IgGs via the Fc region. Various sample preparation strategies can then be used to answer different questions regarding disposition and these strategies can be classified as intact, middle-down, and bottom-up (Figure 5).

### 4.1. Intact Mass Analysis

Measurement of the intact ADC, also known as a top-down approach, can provide information on DAR, glycosylation, and biotransformations, although it lacks site-specific information. The advantage of top-down analysis is that little sample preparation is required, allowing a more high-throughput analysis compared to other MS-based techniques. However, this sensitivity is compromised due to the large size of the ADC (>150 kDa), which, along with its inherent heterogeneity, results in the MS signal being spread across numerous charge states. For this reason, a high-resolution mass spectrometer, such as a time-of-flight (TOF), Orbitrap, or FTICR mass spectrometer is employed for detection of intact ADCs. It is useful to deconvolute the mass spectra to the single-charge domain to identify the various drug forms present. Liquid chromatography is accomplished using a reversed-phase column (e.g., PLRP) with a standard mobile phase comprised of 0.1% formic acid in water and acetonitrile. While structural information from an intact mass analysis is not site-specific, it can provide important structural information to characterize the ADC. For example, Xu et al. were able to monitor the change in DAR in plasma over 21 days after administration of Anti-MUC16 TDC [162]. He et al. monitored the DAR for trastuzumab emtansine 7 days post-dose and cytotoxic drug loss via maleimide exchange through intact mass analysis using a quadrupole-TOF mass spectrometer [163].

### 4.2. Middle-Down

The complexity of mass analysis of an ADC can be reduced and sensitivity improved by performing middle-down analysis, which involves reducing the ADC to its subunits for analysis. IdeS is an enzyme that cleaves the ADC into its Fab and Fc regions. With the use of a reducing agent such as DTT or TCEP, the heavy and light chains can be separated to further simplify analysis. Although the fragments are smaller, a high-resolution mass spectrometer, such as those described above for intact mass measurement, is required. Applications of middle-down quantitation include in vivo and in vitro DAR characterization, drug conjugation site, glycosylation site, and biotransformation characterization. Hernandez-Alba et al. used middle-down mass spectrometry to analyze subunits of a site-specific DAR4 ADC by Orbitrap mass spectrometry, and with the application of various MS/MS methods were able to elucidate drug conjugation and glycosylation sites for the ADC subunits [164].

### 4.3. Bottom-Up

A bottom-up technique, which employs a signature peptide derived from the antibody to represent the entire ADC, provides the most sensitive measurement, but requires the most sample manipulation. Once the ADC is isolated by affinity capture, it is subjected to reduction (e.g., DTT, TCEP), alkylation (e.g., iodoacetamide, chloroacetamide), and digestion using a protease. Trypsin is the most commonly used enzyme as it cleaves at the C-terminal ends of lysine (K) and arginine (R), generating peptides with basic residues that are amenable to ionization in the positive-ion mode. Quantitation is then performed by measuring the signature peptide by LC-MS/MS using selected-reaction monitoring (SRM), typically using a triple quadrupole mass spectrometer.

Much care must be taken in selecting a signature peptide for quantitation. In order for a signature peptide to be selective, it must be unique to the antibody (i.e., selected from the complementarity-determining region) and then confirmed against the human proteome using the pBLAST algorithm. The peptide should not contain a glycosylation site, an amino acid vulnerable to modification (e.g, oxidation of methionine and tryptophan, deamidation of asparagine and glutamine) or a site of attachment for the payload, unless the goal of the measurement is to also obtain occupancy. Furthermore, the peptide must behave well in reversed-phase chromatography and able to produce an abundant signal in the mass spectrum. Open-source software such as Skyline can assist in choosing an appropriate signature peptide [165]. The optimal internal standard is a stable-isotope labeled (SIL) ADC that can go through the entire sample process, but this is not always feasible or cost effective. As an alternative, a SIL version of the signature peptide can be synthesized to serve as the internal standard in the assay.

A bottom-up approach can provide quantitative information on the amount of total antibody, conjugated antibody, and unconjugated drug. An advantage of the bottom-up approach over intact or middle-down is that it can proved site-specific information, such as site conjugation and biotransformations [166]. Chiu et al. have demonstrated the use of a general method to measure IgG1 antibodies from plasma by isolating them with Protein G and measuring signature peptides by LC-MS/MS on a triple quadrupole MS. Hyung et al. developed a method for an ADC with a noncleavable linker where the selected signature peptide contained a cysteine that is linked to the cytotoxic MMAE [167]. Using this approach, the investigators are able to detect the amount of antibody-conjugated MMAE as well as amount of total ADC.

## 5. Managing the Therapeutic Index (TI) of ADCs

A central challenge in the ongoing development of ADCs is the narrow therapeutic index (TI) witnessed in preclinical models and clinical trials. By definition, a way of improving the TI of an oncology agent occurs by increasing the delivery of an agent to malignant cells while avoiding normal healthy tissues. As such, the major elements in TI improvement involve either determining improved methods for selecting which tumors will respond to ADC therapy or improving the delivery of ADCs into the tumor and eventually into tumor cells.

### 5.1. Patient Selection Strategies

Due to the nature of ADCs being meant to delivery highly toxic payloads to malignant cells while sparing normal cells, it is essential to maximize the use of biomarkers as part of clinical trials and the eventual approved dosing guidance to achieve maximum efficacy while minimizing toxicities. However, this approach can be challenging as biomarker-based trials can be complicated during earlier stages of development due to various factors, such as by the biomarker assays being utilized, quality and heterogeneity of samples, and changes in biomarkers due to prior therapies [168]. This is especially a concern when using samples from solid tumors. The various patient selection strategies utilized in clinical trials, which translates into clinical practice and dosing suggestions, each have their own advantages and disadvantages. Thus far, early-stage registration trials of ADCs have mainly focused on patient selection strategies related to target expression, either through a prospective enrollment evaluation or by retrospectively evaluating necessary markers [168]. It is important to weigh the ADC physiochemical factors (e.g., antigen prevalence) and logistical factors (e.g., patient population of interest, biomarker assay limitations) in the selection of the most appropriate selection strategy.

If the target antigen has been well characterized, including expression cut-offs or expression is confirmed with the specific cancer diagnosis, a prospective approach to patient selection may be preferable. This strategy has been applied in the majority of late-stage registration trials for currently approved ADCs (e.g., ado-trastuzumab emtansine, gemtuzumab ozogamicin). However, such a strategy was not commonly employed for ADCs like inotuzumab ozogamicin (anti-CD22) or brentuximab vedotin (anti-CD30) whose target expression has been confirmed to be uniformly expressed at high levels on malignant cells.

Compared to a prospective approach, a retrospective selection design can be used in two situations. The first situation assumes that expression is highly uniform and overexpressed on all malignant cell targets. For instance, polatuzumab vedotin utilized a retrospective strategy in patients with lymphoma as it was determined based on preclinical studies that the target CD79b was expressed by most patients [169]. This assumed homogenous expression within patients was later confirmed in the phase II studies in follicular lymphoma and diffuse large B cell lymphoma where CD79b expression was not associated with tumor growth inhibition [170]. The second situation assumes that a range of the target expression can be evaluated among patients, thus also allowing for exploratory analyses and correlations between clinical outcomes and variation in target expression. Such analyses could allow clinicians to determine theoretical cut-off/threshold values in target expression that result in a meaningful clinical response. Enrichment-based strategies can also be attempted if preclinical or early-stage trials provide insights to a minimum expression profile required to achieve some level of response. However, the greatest risk of a retrospective analysis is that patients are being recruited that may not express sufficient target antigen for ADC efficacy, requiring both logistics and capital funding for the treatment of patients whose data do not benefit the overall study outcomes. As such, retrospective analyses are typically reserved for malignancies with high levels of ADC target expression. Later indications, where expression may be more variable, can be explored once initial methods and data suggests if a target expression threshold correlates with ADC efficacy.

It has been suggested that a retrospective process is also best for evaluating ADCs which have been modified, such as Fc-backbone alterations, structured glycosylation, or change in cytotoxic payload while maintaining a similar antibody backbone, which could allow ADCs to obtain response at lower levels of target expression. An example of this situation can be seen when comparing ado-trastuzumab emtansine compared to trastuzumab deruxtecan. Both of these ADCs utilize the same antibody format and structure but differ in their cytotoxic payload (including the DAR, potency of the cytotoxic agent, and capability to induce a bystander effect). Despite the targeting similarity, trastuzumab deruxtecan appears to be able to obtain responses in patients with HER2 expression at a lower level than that of ado-trastuzumab emtansine, including those below the limit of quantification of the companion diagnostic HercepTest [171,172]. This data also highlights the role minor modifications, such as choice in cytotoxic agent, can have on the effects on the ADC as a whole.

In summary, registration trials are best designed after the ADC target expression is well characterized prior to enrollment to ensure the maximum probability of success when determining patient selection. If the target expression is unknown within patients, this variability should be verified as part of the clinical study. Logistical challenges will exist within trials that use this retrospective approach due to issues in both sample collection and/or sample quality. However, if target expression is found to be stable, a prospective selection process can be achieved using archived samples and improve later during study enrollment. It is possible that improvements in digital imaging with novel quantitative probes or measures will aid in the better understanding of target expression, target heterogeneity, and clinical response. Therefore, patient selection decisions will require weighing the benefits and limitations of each strategy along with patient-specific and disease-specific tumor information in association with the relevant biomarkers. As researchers and clinicians begin to understand how to incorporate ADC and patient biology into achieving improved outcomes (see Section 2 and Section 3), such patient selection strategies will require further development and reassessment but may benefit from enrichment-based strategies.

### 5.2. Optimizing the Delivery of ADCs—Tumor Penetration

A major hurdle that needs to be overcome in optimizing the TI of ADCs is improving the access to the tumor and the tumor-associated antigens recognized by the ADC. The limited efficacy of ADCs can be heavily blamed on the limited penetration into the tumor. While the use of highly cytotoxic warheads is a hallmark of an ADCs, without sufficient overall tumor delivery, these payloads are more likely to result in toxicity than anti-tumor effects. Moreover, the potential for developing toxicities needs to be balanced against evidence that only a small fraction (~0.01%) of the injected dose of an ADC binds to tumor-specific antigen targets [173]. Therefore, additional efforts need to focus on increasing the penetration of ADCs into tumors.

The primary method to increase tumor penetration of ADCs at this time has revolved around making ADCs smaller [49,174]. ADCs are typically designed with full-sized IgG antibodies; however, the large size of an IgG results in a physical barrier or limitation to tumor penetration where smaller sizes of ADCs may improve not be hindered by this barrier [175]. While Fab fragments have been studied for several years without a clinical success, newer methods of making Fab fragments employ non-standard antibody conformations or constructs using smaller binding units, such as mAb scaffolds, diabodies, affibodies, nanobodies, and designed ankyrin repeat proteins (DARPins) [49,174]. Due to the small size of these compounds, they are able to clear rapidly from circulation and normal tissues while facilitating deeper penetration (and binding) within tumor extravascular space [176]. DARPins and other mAb scaffold constructs are unique as these compounds utilize disulfide linkers that selectively release their payloads when delivered and cleaved by tumor microenvironment specific factors [49,174]. The faster clearance of these smaller compounds can also offer an improved toxicity profile, though alternative dosing regimens will likely be required (i.e., administration more frequently than once every 3 to 4 weeks as is used with standard mAbs and ADCs) to achieve anti-tumor efficacy. There have been various small-format ADCs developed to target HER2; however, results of these clinical studies show that these small sized ADCs are better suited for use as theranostics in nuclear medicine due to their high affinity to the target and short biological half-life compared to use as therapeutic agents [177].

**Figure 6:**
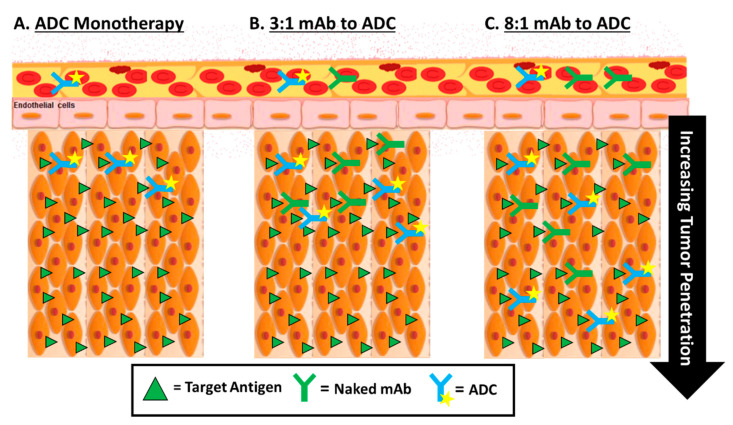
Improving the tumor penetration of ADCs in solid tumors through co-administration of matching non-conjugated (‘naked’) antibody therapy. (**A**) The administration of ADCs as a monotherapy may result in only perivascular distribution into the tumor due to rapid biding of target antigens just outside of the vasculature. (**B**,**C**) In theory, this binding site barrier can be overcome by providing ‘naked’ mAb therapy to compete for target antigen sites—resulting in ADCs to travel further into the tumor to find available antigen to bind. However, administration of the combination of ADC and naked mAb may also inhibit the clearance of the ADC by the IIS/MPS. Thus, the increased delivery of the ADC in combination with mAb may be due an overall higher systemic/serum exposure resulting in the potential for increased delivery into the tumor. The effects of co-administration of ADCs and mAb on saturation of clearance via the MPS needs to be evaluated for each combination regimen.

A second way to improve ADC tumor penetration is to use various methods to overcome the binding site barrier effect. This effect is a phenomenon where the ADC exhibit higher binding affinity for their target antigen located closer to the vasculature instead of further within the tumor, thereby limiting the movement of ADC the further it gets from a blood vessel [1,5]. Consequently, the released drug molecule from the ADC would not be able to kill the cells that they cannot reach. While no clinical studies have been attempted to directly overcome this effect, it is believed that smaller ADCs may display improved permeability without modulating antigen presentation in the tumor. One such example is to attempt to overcome this binding-site effect by coadministration of antibodies, specifically companion ‘naked’ antibodies used in the production of the ADC (Figure 6). Singh et al. conducted in vivo and PK/PD simulation experiments to evaluate the utility of antibody coadministration. Results showed that coadministration strategies improved ADC efficacy with some conditions but did not have an impact on ADC efficacy in others, suggesting that cost and benefit should be analyzed before using this approach [178].

As ADCs appear to exhibit pharmacologic properties similar to nanoparticles, similar studies of the biological mechanisms of tissue diffusion and penetration could yield novel results and ideas to traffic ADCs out of the vasculature. Typically, ADCs are administered a much lower dose in comparison to their parent antibodies due to DLTs caused by the cytotoxic payload, resulting in further decreased penetration into solid tumors. With these lower doses, biodistribution properties of the ADC becomes even more important. ADCs are known to exhibit nanoparticle-like properties where the vascular and lymphatic systems are involved in trafficking. The increased interstitial fluid pressure within tumors limits fluid flow across the vessel wall and instead creates outward fluid motion from the tumor’s periphery, thus reducing the tumor accumulation of antibodies [179,180,181]. The dense extracellular matrix of the tumor also contributes to tissue penetration through creating a physical barrier the ADCs cannot penetrate and causing nonspecific interactions of ADCs [175,182].

## 6. The Next Generation

The growth of ADCs has resulted in a great advancement in the capability to clinicians to selectively battle hematologic malignancies and an ever-growing population of solid tumor malignancies. There are still several challenges for ADCs to overcome, especially as immune-based therapies have taken the spotlight of therapy in oncology. With the regulatory approval of a total of five ADCs in 2019 and 2020, the future of this class of therapeutics is very promising.

Efforts to further optimize ADC therapy through the engineering of unique platforms are in progress. While the success of ADCs has been staggering in a select patient population, ADCs overall still have yet to meet their full clinical promise. However, ongoing efforts to understand the pharmacology of ADCs will help in the engineering of better ADCs. In this review, we have provided a survey of the key factors that can influence an ADC’s disposition. However, certain factors in engineering an ADC still require optimization.

### 6.1. Formulation Strategies

With the approval of ten ADC agents by the FDA, and 80+ ADCs having been evaluated within clinical trials, a wide spectrum of successes and limitations have been reported for ADCs [183]. Among these, most ADCs have been terminated due to unwanted toxicities commonly stemming from the cytotoxic payload [183]. However, these unwanted adverse events in agents stopped in development can help us understand necessary changes in the basic structure and design of an ADC in order to optimize the engineering of the next generation of agents.

#### 6.1.1. Novel Target Antigens

As the current ADC landscape is based primarily around the lysosomal degradation of the ADC, current target antigen optimization relies on efficient internalization after binding a target on the surface of a malignant cell. Because of this, the majority of newer ADC targets are focused on antigen that can be found on the surface of malignant cells or cancer stem cells. While current practices evaluate the level and homogeneity of expression of targets on tumor and normal cells, a factor that is not commonly evaluated until later stage registration trials is the risk of shedding (or secretion) or the target antigen as this can result in increased toxicities [1,5].

Other than using the above criteria for optimal ADC target antigen selection of known receptors (also discussed above in Section 2), the identification of new antigens is an alternate means to develop the next generation of ADC. Considering the success of HER2 targeted therapies for breast and gastric cancers, finding additional targets that synergize with HER2 therapies or brain metastases common within patients with advanced breast cancer that have progressed has been of particular interest. HER3 has been identified as a potentially suitable antigen as this antigen is overexpressed on metastatic brain cancer lesions and may be able to serve as an alternative target for HER2 positive tumors beginning to metastasize [184,185]. While the overall expression of HER3 is lower in comparison to HER2, preclinical studies showed promising efficacy and the results of a phase I/II study in HER3-positive breast cancer showed response rates of ~60% [186,187,188]. Bladder and urothelial cancer have also gained increasing research focus since 2018 and the search for novel antigens to treat these cancers has provided a rich arena for growth [189]. An example of a novel antigen in bladder cancer is the Thomsen-Fridenreich antigen (TF-Ag), a disaccharide hidden on the inside of normal cells but expressed on the exterior of many bladder, colon, and prostate cancers [190]. Preliminary studies have shown this antigen allows for successful mAb binding and internalization, so future studies of ADC conjugates are underway.

Finally, while antibodies are most commonly associated with antigen targeting, ADCs are also being developed to conjugate cytotoxic drugs to tumor-specific receptor ligands. While these targeting modalities are not covered in this review, we point readers to these recently published reviews [191,192,193,194,195].

#### 6.1.2. Novel Antibodies

As a key characteristic of an ADC, and therapeutic mAbs in general, the capability to engineer an agent with enhanced affinity and specificity are of primary importance to improve the pharmacokinetic profile of an ADC. As tumor penetration has also been a key challenge to overcome, ADCs with smaller targeting antibodies have been explored to improve anti-tumor efficacy [196,197,198]. These smaller formats, briefly mentioned above in “Optimizing the delivery of ADCs—Tumor penetration”, include ADCs generated with antibody fragments (e.g., Fab fragments or diabodies) or peptide scaffolds (e.g., DARPin, adnectin) and are still being optimized in preclinical studies [196,197,198,199]. However, while the theory is that smaller ADCs will allow improved penetration, further studies are still necessary to show which of these miniaturized technologies results in enhanced penetration while maintaining anti-tumor efficacy despite an increased systemic clearance [200]. Finally, while data are currently limited, peptide-drug conjugates (agents even smaller than antibody fragments or protein scaffolds) are being developed with the goal to achieve a pharmacokinetic profile similar to traditional small molecule cytotoxic agents in its efficacy while mainlining targeting like an antibody and clearance like a peptide [201,202,203].

One of the novel strategies in antibody optimization is in the use of bispecific antibodies, which are antibodies that contain two different heavy and light chain arms to target two separate target antigens [204]. While their evaluation is still mainly in the preclinical stages, there have been several promising studies to prove the concept [205,206]. One of the most cited examples of the application of bispecific antibodies are studies of a HER2-Prolactin Receptor (PRLR) bispecific ADC [207]. This study was able to demonstrate how breast cancer cells that expressed both antigens were killed more efficiently using the bispecific antibody compared to a HER2-DM1 ADC or PRLR-DM1 ADC alone [207]. Another form of bispecific antibody being evaluated in next generation ADCs are biparatopic antibodies, or an antibody that recognizes two different binding epitopes (that are not over-lapping) on the same target antigen [208]. This technology has been successfully applied to HER2 targeting antibodies as clustering of HER2 can result in increased internalization, potentially improving ADC performance [209]. Though the design of a biparatropic mAb which targets the epitopes for the existing therapeutic mAbs trastuzumab and pertuzumab, a combination already utilized in the treatment of late-stage breast cancer, has been discontinued due to toxicities, an alternative antibody targeting the trastuzumab epitope and a novel HER2 epitope is being evaluated [210].

A final novel antibody approach is in the use of nanobodies (i.e., small immunoproteins [SIPs] and single-chain variable fragments [scFv]) [211,212]. These antibodies are currently primarily derived from camelids (e.g., camels, llamas, alpacas) which utilize a unique heavy-chain only antibody conformation [211,213]. Other than the capability for natural production, nanobodies also offer the benefit of water solubility with high formulation stability while preserving strong antigen affinity [214]. As nanobodies also bind epitopes in a fashion that does not impact traditional antibody binding and can be engineered to be isoform specific, this antibody platform has high potential to generate highly-specific diagnostic companion agents [215,216]. While there are no currently approved ADCs using nanobodies, caplacizumab (Cablivi) was the first nanobody approved in 2019 by the FDA for the treatment of thrombotic thrombocytopenic purpura (TTP) [217].

#### 6.1.3. Novel Payloads

All of the FDA approved ADC agents use one of two mechanisms of action (discussed previously in “Payload options”). However, with the advent of higher DAR technologies and mechanisms to stabilize ADCs, alternative payloads can now be utilized. The focus of these new payloads has mainly been to provide additional mechanisms of action that can overcome acquired multi-drug resistance from prior ADC therapies. While several novel cytotoxic payloads have also been discussed in the above “Payload options” section (e.g., amanitins, Bcl-xL inhibitors, camptothecin analogs), two emerging payloads have also begun to show promise.

The first of these payloads is the conjugation of nitric oxide (NO) donors to ADCs. The only example of this payload strategy has been presented by Fumou et al., where a CD24-NO donor agent was synthesized and incubated in vitro or administered in mice with hepatocellular carcinoma cells [218]. The CD24-NO ADC resulted in improved efficacy compared to the antibody or payload being administered alone. However, while NO has been well characterized within in vitro and in vivo models to inhibit tumor progression, the same mechanisms can stimulate tumor proliferation as well [219]. Because of this, we could expect a wide variability in patient response and response could be improved with concomitant therapy with NOS inhibitors to reduce endogenous NO production.

Finally, gene therapies using RNA interference (RNAi) have now progressed to the point where they can be successfully applied to treat various diseases, including cancers [220]. These payloads, consisting of noncoding double-stranded RNA chains, work by degrading messenger RNA (mRNA) and preventing the translation of target proteins [221,222]. This makes RNAi technologies innately superior in inhibiting protein targets compared to other targeted small molecule inhibitors. The main issue with RNAi therapies is to be able to deliver the RNA strands safely to the interior of malignant cells without degrading or being enzymatically broken down. While other technologies have done this by using polymers and various nano-lipid preparations, these preparations lack a mechanism of tissue-specific delivery—a limitation that is not a concern for ADCs [223]. As encapsulation techniques cannot be utilized with ADCs, the translation of RNAi probes under physiologic conditions via chemical modification are still necessary to make this payload a viable ADC strategy [224]. However, recent novel conjugations of small-format antibodies to cytotoxic drugs along with aptamers or genes (such as ‘suicide genes’) have emerged, generating compounds with two potential therapeutic properties to exert anti-tumor activity [212,225].

### 6.2. Therapeutic Strategies

Other than altering the structures that make up the current strategies of ADC production, new and innovative strategies are being developed to change the therapeutic strategy and approach to ADC administration.

*Targeting the Tumor Microenvironment.* While the main mechanism of action for ADCs relies upon internalization of ADCs to release the cytotoxic payload to kill both the internalizing malignant cell and surrounding cells (i.e., the bystander effect), the rate of internalization by tumor cells is variable and thus potentially highly inconsistent within tumors and between patients. In order to normalize and potentially increase the death of malignant cells, newer theories and strategies have sought to be able to release the cytotoxic payload in the vicinity of large groups of tumor cells without requiring internalization [226,227,228]. As the various components of the tumor microenvironment (TME) have also been shown to play an important role in tumor growth and metastasis, instead of targeting tumor specific antigens, targeting antigens of the TME will allow ADC to accumulate within tumors and release their payload based on TME-specific factors [226,227].

A different tactic is to target the immunosuppressive cells (e.g., regulatory T cells) within the TME to overcome their suppressive effects and make tumors more immune competent [229,230,231,232]. Finally, while therapeutic mAbs targeting PD-L1 have provided robust anti-tumor effects in a subset of patients, the use of PD-L1 targets in the TME are also being explored as a PD-L1 ADC could provide both immune cell activation and delivery of cytotoxic payloads to the TME extravascular space (as PD-L1 is not internalized into cells upon binding) [233]. The targeting of TME-associated antigens may also be of greater benefit as these targets are likely more accessible from the vasculature [234]. However, just like tumor-associated antigens, effort should be made to ensure TME targets are not (or minimally) expressed in normal tissues. While this may seem equally disadvantageous to previously developed ADCs, the targeting of the TME may allow for a single ADC to carry indications within multiple solid tumors (not being limited to a tumor-specific antigen) and the risk for developing resistance is decreased (as normal tissues do not undergo somatic mutations as easily to develop drug resistance). In addition, ADCs targeting tumor-specific secreted extracellular proteins (e.g., Gal-3BP) formed by tumors cells are becoming a new target of interest [235,236]. The goal is that these extracellular protein targets are selectively localized around tumors and not normal cells, therefore aiding non-internalizing ADCs with a higher bystander killing activity.

#### 6.2.1. Combination Therapy

A lesson every clinician and researcher in oncology has learned is that various forms of resistance to given therapies will develop. Because of this, the investigation into combination therapy utilizing agents with differing mechanisms of action can aid in providing synergistic anti-tumor killing effect and reducing the opportunity for resistance or metastasis to occur—all resulting in improved cancer survival rates [237]. This is of even better benefit as early findings suggest that combining ADCs with other drugs has led to both improved efficacy and reduced toxicity of ADCs [2]. The key example of this combination of agents is a study by Boshuizen et al., where an AXL-107-MMAE ADC was combined with a small molecule BRAF/MEK inhibitor [238]. This may be a unique and well-designed example, as the combination of these targeted agents both targeting mechanisms tumor utilize to develop drug-resistance, as combinations with other traditional small molecule cytotoxic agents may not provide as desirable toxicity profiles. In addition, due to the early success of therapeutic mAbs, especially with the PD-1 and PD-L1 checkpoint inhibitors, combinations are being explored. Early preclinical results have demonstrated that the combination for checkpoint blockade inhibitors (CBI) could increase the tumor penetration of CD8+ T cells in order to improve the anti-tumor immune response alongside ADC cytotoxic killing of malignant cells. There are a number of phase I and phase II studies currently recruiting studies with actively approved ADCs (mainly ado-trastuzumab emtansine or brentuximab vedotin) with various CBI therapies (pembrolizumab, atezolizumab, ipilimumab, nivolumab) [239,240,241,242,243].

#### 6.2.2. Conditional Activation

A unique approach entering the field is to have the capability to regulate non-specific interaction/uptake of the ADC by making their activation conditional based on tumor-specific influences [244,245]. This novel approach, currently termed as conditionally activated biologics (CAB), could allow for expanded use of previous target antigen that are also found on non-malignant tissues as activation of the ADC would be limited unless the tumor-specific influences were present. Based on this theory, taking advantage of the TME will play a critical role in the success of these agents in conjugation with ensuring adequate target antigen binding and penetration. One mechanism for CAB agents is to engineer the antibody to bind their target antigen with a greater affinity when in the presence and to decrease their affinity when the target is further away [246,247,248,249]. This mechanism is being evaluated in two agents currently in phase I trials, BA3011 (which targets AXL) and BA3021 (which targets ROR2) [246,247,248,249]. The activation of these antibodies depends on the pH of the local environment; lower pH (~pH 6) induces a conformation with greater affinity to the target epitope while higher pH (~pH 7.4) will decrease the binding affinity. A second CAB mechanism makes use of a probody to mask the main antigen binding region of the ADC upon administration and distribution, though ultimately degraded by TME-residing proteases (e.g., urokinase, matriptase) to activate ADC target binding [250,251,252]. Two agents utilizing this CAB mechanism are currently in phase I trials: CX-2009 (CD116-DM4 ADC) and CX-2029 (CD71-MMAE ADC) [253,254].

### 6.3. Avoiding Resistance

Just as with any other therapeutic strategy, the development of resistance to ADCs will be a key issue to overcome, especially as more agents begin to enter the clinic. While more classic forms of resistance can occur, such as alterations in transporters affecting certain cytotoxic warheads or the development of anti-drug antibodies, recent evidence has presented that the mechanisms of resistance of ADCs are highly heterogenous [103,255,256,257]. A well-documented example of this is the resistance of ado-trastuzumab emtansine, where resistance showed both a down regulation in the expression of HER2 (the target antigen) and expression of multi-drug resistance genes [103]. However, a unique aspect to ADC resistances is that the resistance to a single agent does not appear to translate to other ADCs with the same target antigen so long as the conjugated payload utilizes a different mechanism of action [103]. This creates a unique line of clinical investigations to see how well patients failing one ADC could be recovered using a similar ADC, such as trastuzumab deruxtecan can salvage those resistant to ado-trastuzumab emtansine. Ultimately, more clinical evidence and investigations into the types and combinations of resistances observed in patients are still pending. Once such knowledge is obtained, appropriate models can then be developed to aid pharmacologists overcome or prevent resistance.

### 6.4. ADCs as Immune Modulators

One of the more novel applications of ADCs technology has not been to delivery cytotoxic agents, but delivery immune-stimulants to provide anti-tumor immunity. The most common mechanism of action has been to use agents that activate APCs and therefore providing a synergistic effect with checkpoint blockade [258]. An example of this new age ADC is BDC-1001, a HER2 targeted mAb conjugated to a TLR7/8 agonist, which is being evaluated with and without combination of pembrolizumab in patients with breast or gastric malignancies [259,260]. The premise of this combination is that the ADCs will localize around HER2-positive tumor cells; once bound, the free Fc-region of the ADC can interact with macrophages and dendritic cells, effectively delivering the TLR agonist to induce direct macrophage killing of the malignant cells and promote tumor-specific lymphocyte response [259]. These ADCs have been highly promising as preclinical studies have shown little evidence of systemic toxicities, especially in comparison to traditional cytotoxic chemotherapies.

### 6.5. Therapeutic Drug Monitoring

Several analyses from early-stage trials have identified various factors with the potential to influence the disposition of mAbs and ADCs. As these factors have a direct effect on the PK of ADCs, therapeutic drug monitoring (TDM) has been proposed to evaluate and respond to the inherent inter-patient variability observed within patients. However, these efforts are hindered due to the complex pharmacology of these agents, including nonlinear distribution and elimination [261,262,263]. While significant progress has been made in our knowledge and understanding of the PK and PD of ADCs, further probing of the mechanistic aspects of their disposition and how this relates to efficacy and safety is still needed.

Furthermore, due to the multiple forms an ADC can take in circulation (ADC, unconjugated mAb, unconjugated cytotoxic payload), additional efforts will be needed to characterize each form. These characterizations are necessary to determine concentrations of greatest relevance to reflect anti-tumor efficacy or toxicity and patient-specific factors (e.g., serum albumin) effect on their individual forms. Several studies have demonstrated how serum trough concentrations of therapeutic mAbs correlate with a therapeutic response, though more extensive analyses are necessary for ADCs to achieve a similar level of evidence [146,264,265,266,267,268,269,270]. However, ADC therapies also present with new toxicities that are associated with enhanced distribution to specific organs (e.g., liver) and toxicities associated with the components of the carrier.

Similarly, a great effort can still be done to improve the dosing of ADCs using TDM strategies. The idea of using patient-specific factors to personalize ADC therapy for a patient in order to make therapy more effective (and potentially less costly). Previous studies and reviews have shown how different properties (physical characteristics, host-associated factors, pharmacologic interactions, and interactions with the MPS) all contribute to the altering the PK/PD of these agents in both preclinical models and patients [1,5]. While limited attempts at TDM have been attempted with ADCs, both dose and dosing interval are likely variables that will need to be adjusted in tandem to achieve optimal therapeutic outcomes. Based on prior data from other therapeutic mAbs, such as infliximab, the use of Bayesian and individual population PK models could both provide a pathway to incorporate such factors know to influence mAb PK (e.g., serum albumin, ADA, MPS activity/features, body weight) [271,272].

### 6.6. Optimized Dosing Based on Biomarkers and Precision Medicine Methods for Individual Patients or Selected Patient Groups

There has been a flood of different technologies and several biomarkers being explored for immunotherapies with potential as predictive markers of on-treatment therapeutic success. While markers such as high mutational burden appear to correlate with a high likelihood of response for checkpoint blockade therapies, the capability to precisely define markers which guide response remains elusive. Based on the current literature, the analysis of tumor biopsies obtained while early on-treatment seems to offer the most effective method of monitoring the effective engagement of both immunity and tumor response. However, the feasibility of this type of monitoring strategy is questionable as this evidence was found through serial tumor biopsies—a tactic which would not translate well into routine clinical practice, especially those with advanced cancers. Because of this, strategies that focus on blood-based markers have begun to gain more traction. In some cases, these blood-based markers have been shown to demonstrate direct evidence of immune activation in tumors. As the current portfolio of immunotherapy trials exceeds the available pool of trial participants, detecting response and resistance to available immunotherapies, including ADCs with or without combinations of other immune-modulating therapies, would accelerate proof-of-concept studies, allowing for prioritization of agents and selection of secondary agents at the time of disease progression to study further. In addition, the use of biomarkers in the blood of patients (e.g., MPS FcɣRs) is a novel potential method to optimize the dose and improve the therapeutic index of mAbs and ADCs for individual patients or selected patient groups.

## 7. The Next Generation

The promises provided by immunotherapies in the treatment of cancer have resulted in sustained responses and remission in a select subset of patients. The next progression forward with the advent of ADCs sought to improve these initial response rates. While the stories of the approval of the first ADCs like ado-trastuzumab emtansine and brentuximab vedotin provide numerous stories of the success of these novel engineered agents, there are equal numbers of troubles encountered, such as those of the first release of gemtuzumab ozogamicin, highlighting that our understanding of the pharmacology of ADCs is still incomplete. However, by understanding the factors that affect the clinical application of these biologic therapies the approach and strategies to engineering the next generation of ADCs have already begun to show improvements. The continuing development and exploration into new antibody formats, antigens, and cytotoxic payloads will require a greater emphasis in basic and preclinical research models before translation to an appropriate patient population. As ADCs are being used more frequently in clinical practice, an improvement in the understanding of the pathways involved in resistance to ADC therapies can provide further information on rational combination therapies and better treatment outcomes. Combining these data with advancements in clinical trial recruitment and design, ADCs could begin to envision a clear and accelerated path to approval.

## Figures and Tables

**Table 1:** Antibody-drug conjugates approved by the FDA.

Generic Name	Brand Name (Manufacturer)	Indication	Approval Year	ADC Characteristics			
				Antibody Isotype	Target Antigen	Linker	Payload
Brentuximab vedotin	Adcetris (Seattle Genetics)	HL, ALCL	2011	IgG1	CD30	Cleavable (protease)	MMAE
Ado-trastuzumab emtasine	Kadcyla (Genentech)	HER2+ mBC HER2+ eBC	2013	IgG1	HER2	Non-cleavable	DM1
Inotuzumab ozogamicin	Besponsa (Pfizer)	ALL	2017	IgG4	CD22	Cleavable (acid labile)	Calicheamicin
Gemtuzumab ozogamicin	Mylotarg (Pfizer)	CD33+ AML	2017	IgG4	CD33	Cleavable (disulfide)	Calicheamicin
Moxetumomab pasudotox-tdfk	Lumoxiti (AstraZeneca)	r/r HCL	2018	IgG1	CD22	Cleavable (disulfide)	*Pseudomonas* exotoxin
Polatuzumab vedotin	Polivy (Genentech)	r/r DLBCL	2019	IgG1	CD79b	Cleavable (protease)	MMAE
Enfortumab vedotin	Padcev (Seattle Genetics)	mUC	2019	IgG1	Nectin-4	Cleavable (protease)	MMAE
Trastuzumab deruxtecan	Enhertu (AstraZeneca) (Daiichi-Sankyo)	HER2+ BC HER2+ GC/GEJ	2019	IgG1	HER2	Cleavable (peptides)	DXd
Sacituzumab govitecan	Trodelvy (Imunomedics)	mTNBC	2020	IgG1	Trop-2	Cleavable (acid labile)	SN-38
Belantamab mafodotin-blmf	Blenrep (GlaxoSmithKline)	Multiple myeloma	2020	IgG1	BCMA	Cleavable (protease)	MMAF

Abbreviations: ALCL, aplastic large cell lymphoma; ALL, acute lymphoblastic leukemia; AML, acute myeloid leukemia; BC, breast cancer; DLBCL, diffuse large B cell lymphoma; DM1, mertansine; Dxd, topoisomerase I inhibitor; eBC, early breast cancer; GC, gastric cancer; GEJ, gastroesophogeal junction adenocarcinoma; HCL, hairy cell lymphoma; HL, Hodgkin lymphoma; mBC, metastatic breast cancer; mc, maleimidocaproyl; MMAE, monomethyl auristatin E; MMAF, monomethyl auristatin F; mTNBC, metastatic triple-negative breast cacner; mUC, metastatic urothelial cancer; r/r, relapsed/refractory; SN-38, active metabolite of irinotecan.

## Data Availability

Not applicable.
